# *Onchocerca armillata* contains the endosymbiotic bacterium *Wolbachia* and elicits a limited inflammatory response

**DOI:** 10.1016/j.vetpar.2010.08.031

**Published:** 2010-12-15

**Authors:** Joseph M. Neary, Alexander J. Trees, David D. Ekale, Vincent N. Tanya, Udo Hetzel, Benjamin L. Makepeace

**Affiliations:** a Liverpool School of Tropical Medicine and Faculty of Veterinary Science, University of Liverpool, Liverpool, UK; b Institut de Recherche Agricole pour le Développement, Wakwa, Adamawa Region, Cameroon

**Keywords:** Bovine, *Onchocerca armillata*, *Wolbachia*, Filariasis, Onchocerciasis

## Abstract

Human onchocerciasis, also known as River Blindness, is a debilitating disease caused by the filarial nematode *Onchocerca volvulus*. Many, but not all, filarial nematodes carry within their tissues endosymbiotic, *Rickettsia*-like bacteria of the genus *Wolbachia*. *Onchocerca* spp. infections in cattle offer the most relevant, analogous host–parasite model system. West African cattle are commonly co-infected with four *Onchocerca* spp.; two of these are *Wolbachia*-positive (*Onchocerca gutturosa* and *Onchocerca ochengi*), and the remainder are of unknown *Wolbachia* status (*Onchocerca dukei* and *Onchocerca armillata*). Previous studies have suggested that worm survival is dependent on this bacterium. *O. armillata*, an abundant parasite of African cattle that has received little attention, is a primitive species that may lack *Wolbachia*. The objectives of this study were to determine if *O. armillata* carries *Wolbachia* and to provide preliminary descriptions of the host inflammatory cell environment around the adult worms. The findings may support or refute the hypothesis that a prime contribution of *Wolbachia* is to permit long-term survival and reproduction of certain *Onchocerca* spp. (including *O. volvulus* in humans). *O. armillata* adult worms were found in the aorta of 90.7% of cattle (*n* = 54) slaughtered at an abattoir in Ngaoundéré, Adamawa Region, Cameroon. The presence of *Wolbachia* in *O. armillata* was confirmed by a specific anti-*Wolbachia* surface protein antibody detected using a peroxidase conjugate (immunohistochemistry) and PCR for detection of *Wolbachia*-specific sequences within DNA extracts from frozen worms. Tissue sections stained with haematoxylin and eosin showed the host cell response to be dominated by macrophages and fibroblasts. This is unusual compared with nodule-dwelling *Wolbachia*-positive *Onchocerca* spp., where the host response is typically characterised by granulocytes, and suggests that the mechanisms for worm survival employed by this species (which is probably motile) may differ.

## 1 Introduction

Filarial nematodes are major pathogens that are responsible for debilitating diseases in human populations of the tropics (‘River Blindness’, caused by *Onchocerca volvulus*; and lymphatic filariasis or elephantiasis, caused by *Wuchereria bancrofti* and *Brugia* spp.) and in animals (canine heartworm, caused by *Dirofilaria immitis*). An estimated 37 million people are infected with *O. volvulus*, with a total of 90 million at risk of infection in Africa (Anon., 2005). The adult female worms reside in subcutaneous nodules within which they must be fertilised by migrating males before they can release microfilariae (Mf) into the surrounding tissue. The Mf accumulate in the skin and eyes, and may be transferred to a female *Simulium* blackfly during a bloodmeal, within which they develop after two moults into infective larvae (L3). The host response to dead and dying Mf may cause immune-mediated alterations, principally severe dermatitis and visual impairment (Enk, 2006).

Control of onchocerciasis is almost exclusively dependent on annual or semi-annual mass administration of ivermectin to the affected communities (Molyneux, 2005). Ivermectin kills the Mf and thus prevents pathology, but fails to kill the adult worms, which may live for >10 years. Currently, there is no macrofilaricidal (*i.e.*, lethal to adult worms) drug that is suitable for mass distribution.

Many, but not all, filarial nematodes carry within hypodermal and other cells endosymbiotic, *Rickettsia*-like bacteria of the genus *Wolbachia*. The relatively recent rediscovery of their presence after their initial description over three decades ago (Kozek and Marroquin, 1977) has stimulated research to exploit them as a drug target (Hoerauf et al., 1999; Langworthy et al., 2000; Bazzocchi et al., 2008); to investigate their possible role in aetiology of filarial disease (Taylor et al., 2000; Saint André et al., 2002); and to determine their functional relationship with their host worms (Fenn and Blaxter, 2004).

The vast majority of species within the genus *Onchocerca* are parasites of ungulates, not primates, and no *Onchocerca* sp. naturally parasitizes rodents. This has led to the development of *Onchocerca ochengi*, a parasite of cattle in sub-Saharan Africa which is the closest relative of *O. volvulus* (Morales-Hojas et al., 2006), as a natural model of human onchocerciasis [see Trees (1992) for review]. It has been shown unequivocally that antibiotic treatment of cattle infected with the *Wolbachia*-positive *O. ochengi* kills adult worms and this is a result of the prior, sustained depletion of *Wolbachia*, suggesting that worm survival depends on this bacterium (Langworthy et al., 2000; Gilbert et al., 2005). Subsequently, clinical trials of doxycycline chemotherapy for human onchocerciasis have demonstrated significant macrofilaricidal activity against *O. volvulus*, although 4–6 weeks of daily treatment were required (Hoerauf et al., 2008).

Sequencing of both filarial and *Wolbachia* genomes in *B. malayi* has revealed possible gene products unique to one or other of the symbiotic partners, which may form the basis of their mutualistic relationship (Foster et al., 2005; Ghedin et al., 2007). Whilst this suggests that the provision of an essential metabolic component may explain worm death following *Wolbachia* depletion, sequential studies *ex vivo* of *O. ochengi* nodules during antibiotic treatment have led us to hypothesise that *Wolbachia* may aid long-term worm survival by preventing eosinophil attack (in otherwise competent hosts) by creating a neutrophil-dominated cellular environment around the worms (Nfon et al., 2006). It is a striking characteristic of both *O. ochengi* and *O. volvulus* (which also contains *Wolbachia*) that they survive and reproduce for many years surrounded by specific antibody and host inflammatory cells dominated by neutrophils (Brattig et al., 2001; Nfon et al., 2006).

Apart from studies on *O. ochengi*, the hypothesis is circumstantially supported by observations on a *Wolbachia*-negative *Onchocerca* of deer, *Onchocerca flexuosa*, in which the lifespan appears short and the cellular environment is dominated by eosinophils and giant cells, in contrast with a *Wolbachia*-positive sympatric species in deer, *Onchocerca jakutensis* (Plenge-Bönig et al., 1995). Deer parasites are difficult to study, but in the most comprehensive phylogenetic analysis of the genus *Onchocerca* published to date (Krueger et al., 2007), *Onchocerca armillata* was considered to represent an ancient, ‘primitive’ lineage that clustered in a basal position alongside *O. flexuosa*. This raises the intriguing possibility that it, too, lacks *Wolbachia*. West African cattle are commonly co-infected with four *Onchocerca* spp.; two of these are *Wolbachia*-positive (*Onchocerca gutturosa* and *O. ochengi*), and the remainder are of unknown *Wolbachia* status (*Onchocerca dukei* and *O. armillata*). In previous abattoir studies, it was noted that whilst *O. armillata* adult worms are very common (they lie in the sub-endothelium of the aorta), they are frequently calcified, and microfilarial prevalence and intensity in the skin is low (Trees et al., 1989). This would be consistent with a short lifespan.

The possibility that West African cattle may be infected with a *Wolbachi*a-negative *Onchocerca* sp. concurrently with the *Wolbachia*-positive *O. ochengi* opens up exciting possibilities for comparative studies in the same accessible host species, in order to support or refute the hypothesis that a prime contribution of *Wolbachia* is to permit long-term survival and reproduction of certain *Onchocerca* spp. (which include *O. volvulus* in humans). In this study, evidence is provided to show that *O. armillata* does contain the endosymbiont *Wolbachia* and a cellular response is described that differs somewhat from other *Wolbachia*-containing *Onchocerca* spp.

## 2 Materials and methods

### 2.1 Study area and sample collection

Samples were collected from cattle reared in the Adamawa highlands of north Cameroon and slaughtered at the abattoir of Ngaoundéré (7°13′N, 13°34′E). This region is 1000 m above sea level and characterized by Guinea savannah vegetation with a single dry (November–March) and rainy season (April–October) in a year. Animal age was estimated by dentition (Kahn, 2005). The aortic arch was examined for evidence of *O. armillata* adult worms and 49 positive specimens were collected. In addition, skin samples of 3–5 cm diameter were taken from the hump and ventral midline (between the udder/scrotum and umbilicus) of all positive animals and one *O. armillata*-negative cow. The age and sex of all animals sampled for aortic infection (irrespective of the presence or absence of *O. armillata*; *n* = 54) was recorded. Within a few hours of slaughter, the aortas and skin samples were dissected and examined at the Institut de Recherche Agricole pour le Développement (IRAD), Regional Centre of Wakwa (approximately 10 km from Ngaoundéré).

### 2.2 Microfilariae

After shaving each skin sample (hump and ventral midline), three slices of superficial skin (mean wet mass per slice, 89 mg) were taken with a scalpel from separate locations on each original sample. These skin slivers were subsequently incubated at 37 °C in Roswell Park Memorial Institute (RPMI) 1640 medium (Lonza, Wokingham, UK) for 6 h, after which the medium was changed and incubation continued overnight for a total of 24 h. The number and species of the emerged Mf present in the medium was determined with a stereo-microscope (at 50× magnification) and confirmed, as required, with a compound microscope.

Microfilariae of *O. armillata* were differentiated from co-infecting *Onchocerca* spp. by longer length (350–400 μm), kidney-shaped appearance when dead, and the prominent cephalic inflation. All four species also have characteristic movement patterns (Wahl et al., 1994).

### 2.3 Immunohistochemistry for *Wolbachia* surface protein (WSP)

Immunohistochemistry for the visualisation of *Wolbachia* was performed on one nodule and four aorta sections (each from a different animal) using a rabbit polyclonal antibody against recombinant *Wolbachia* surface protein (WSP) derived from *D. immitis* (generously donated by M. Casiraghi, University of Milan, Italy). The protocol was originally developed by Kramer et al. (2003) and was subsequently modified by Gilbert et al. (2005). Formalin-fixed, paraffin-embedded tissue sections were ‘demasked’ by immersion in 0.1 M sodium citrate buffer, pH 6.0, and heated at minimum power in a microwave oven (Hinari, LifeStyle, 800W) for two 1-min cycles with a change of buffer. Blocking of endogenous peroxidase and detection of bound antibody was performed using the Universal LSAB2 Horseradish Peroxidase Kit (DakoCytomation, Ely, UK) according to the manufacturer's instructions. This system uses a biotinylated secondary antibody that forms a complex with peroxidase-conjugated streptavidin, which then reacts with a chromogen [3,3′-diaminobenzidine (DAB)], leading to a brown coloured precipitate. To prevent non-specific binding of antibodies, slides were also blocked for 30 min with 1% bovine serum albumin (BSA) and 5% sucrose in wash buffer [10 mM Tris-hydrochloride, pH 8.5; 150 mM sodium chloride, 0.1% (v/v) ‘Tween’-20]. Slides were subsequently incubated for 30 min with anti-WSP primary antibody at a dilution of 1/500 in blocking solution. Adjacent sections cut from the same block were incubated in parallel with normal rabbit serum (Sigma) at the same dilution (negative control). Sections cut from an *O. ochengi* nodule, a species already known to contain and stain for WSP (Gilbert et al., 2005), were developed using the same protocol (positive control). Sections were counter-stained with Harris haematoxylin (HD Supplies) and mounted using DPX. Slides were photographed on a Microphot-FX digital microscope (Nikon, Tokyo, Japan).

### 2.4 PCR

Small sections of dissected aorta were left in PBS solution for 6 h at ambient temperature. This promoted the adult worms to emerge sufficiently from their tunnels to aid extraction from the aorta wall by applying gentle traction on the worm with forceps. The adult worms were immediately frozen at −80 °C and subsequently transported to the UK on dry ice.

The worms were thawed and finely chopped with a scalpel blade, and genomic DNA was extracted from the macerate using DNAzol^®^ reagent (Invitrogen, Paisley, UK) according to the manufacturer's protocol. In order to visualise the DNA pellets and so minimise losses during washing, 2 μl of Pellet Paint^®^ co-precipitant (Novagen^®^, VWR International) was added to the homogenate prior to ethanol precipitation. DNA pellets were dissolved in 30 μl of 8 mM sodium hydroxide and stored at 4 °C.

Published oligonucleotide sequences with broad specificity for the 16S rRNA (Casiraghi et al., 2001) and *ftsZ* (Werren et al., 1995) genes of *Wolbachia* were used for custom primer synthesis (Sigma-Genosys, Haverhill, UK). For both assays, the reaction composition was 1 U Thermo-Start™ *Taq* DNA polymerase, 200 μM each dNTP and 1.5 mM magnesium chloride in 1× High Performance Buffer (all supplied by Abgene, Epsom, UK), with 1 μM each primer and 1 μl DNA template in a final volume of 20 μl. Cycling parameters comprised enzyme activation (95 °C for 15 min), followed by 30 (16S rRNA) or 35 (*ftsZ*) cycles of denaturation (94 °C for 45 s), annealing (52 °C for 45 s) and extension (72 °C for 90 s) on a T3 thermocycler (Biometra^®^, Göttingen, Germany). The expected PCR product size was 1000 base-pairs (bp) for both assays.

For visualisation of PCR products, 5× DNA loading buffer (Bioline, London, UK) was added to the PCR reaction and 5 μl was loaded on a 1% agarose gel containing SYBR^®^ Safe nucleic acid stain (Invitrogen). Electrophoresis in 1× Tris–acetate–EDTA buffer was conducted for 1 h at 100 V, and the gel was imaged using a Safe Imager™ blue-light transilluminator (Invitrogen) and a gel documentation system with GeneSnap software (Syngene, Cambridge, UK).

### 2.5 Histopathology

Tissues fixed in 10% neutral-buffered formalin were cut into 4 μm sections and stained with Mayer's haemalum and eosin. Slides were examined for the integrity of worm sections and the immune response, and photographed on a Microphot-FX digital microscope (Nikon). One nodule was taken from each of 15 cattle of a broad age range and examined. Of these 15 cattle with nodules, 7 sections of aorta wall were also examined. Two slides, one from the nodule of a 3-year-old female and the other from the aorta wall of a female of at least 10 years of age, were examined for Fe^2+^, Ca^2+^ complexes, and endothelial cells with Prussian blue, von Kossa, and Factor VIII-related antigen stains, respectively. This allowed visualisation of Fe^2+^ in haemosiderin from ingested erythrocytes if present, Ca^2+^ in mineralised tissue, and the location of *O. armillata* adult worms with respect to the endothelial-lined microvasculature and lymphatic vessels.

### 2.6 Statistical analysis

All statistics were performed in PASW Statistics 17.0 (SPSS Inc., Chicago, IL, USA). Frequency data were analysed by Fisher's exact test, whereas medians of independent count data were compared using the Mann–Whitney *U*-test or the Kruskal–Wallis test. Paired data were subjected to the log_10_(*x* + 1) transformation to normalise the distribution, and analysed using a paired *t*-test. The critical probability for statistical significance was *P* < 0.05.

## 3 Results

### 3.1 Prevalence of adult *O. armillata* and aortic nodules by host age and sex

Adult worms of *O. armillata* were found in 90.7% (49/54) of the cattle examined (Table 1). Of those animals positive for adult worms, 65.3% (32/49) had nodules in the aorta wall (Table 1). The sampled animals were divided into four predetermined age categories, and the prevalence of adult parasites and nodules was analysed across the age distribution. There was no significant association between age and either the presence of adult worms or aortic nodules (*P* > 0.05, Table 2). Only 6 bulls were examined in this study, reducing the power of any analysis by sex. Although the prevalence of nodules was twice as high in females (69.8%) than in males (33.3%), this was not statistically significant (Fisher's exact test, *P* = 0.16).

### 3.2 Prevalence of *O. armillata* Mf by host age and sex

Only 24.5% (12/49) of the animals with adult parasites in the aorta had detectable patent infections (*i.e.*, Mf in skin samples). The geometric mean density of Mf per 100 mg skin (including zero counts) was 0.23 for hump samples and 0.07 for ventral samples, although this difference was not statistically significant (*P* = 0.10, paired *t*-test). Therefore, the arithmetic mean of data from the two sites for each animal was used in subsequent analyses. There was no significant association between host age and either the prevalence or density of Mf (Table 1). Furthermore, the prevalence of patent infection appeared to be similar between the sexes (16.7% for males, 25.6% for females) and was not statistically significant (Fisher's exact test, *P* = 1.0). The median density of Mf was also not significantly different between the sexes (Mann–Whitney *U*-test, *P* = 0.62).

### 3.3 Microfilariae of other species and predilection sites

*Onchocerca armillata* Mf were considerably less prevalent than both *O. gutturosa* and *O. ochengi* Mf in the study population (Table 2), and Mf densities for *O. armillata* were the lowest of the four *Onchocerca* spp. present (even after exclusion of zero counts). Unlike *O. ochengi*, which exhibited a strong predilection for the ventral midline, *O. armillata* showed only a non-significant trend towards higher Mf densities in the hump (Table 2). Of the twelve animals with a detectable patent infection with *O. armillata*, only one (a 4-year-old female) had a positive Mf count in both the hump and ventral midline.

### 3.4 Immunohistochemistry for *Wolbachia* surface protein (WSP)

**Fig. 1:**
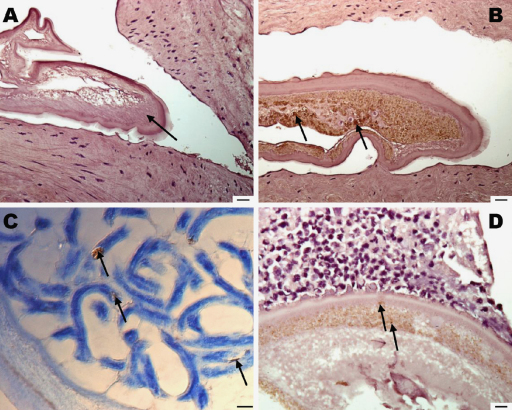
Staining for *Wolbachia* surface protein (WSP) in adult female worms. (A) *O. armillata* negative control (normal rabbit serum) with absence of punctate staining in the hypodermal chords (arrow). (B) *O. armillata* probed with anti-WSP antibody; note heavily stained groups of bacteria in the hypodermis (arrows). (C) *O. armillata* intrauterine microfilariae showing small clusters of stained *Wolbachia* (arrows). (D) *O. ochengi* positive control; note punctate staining of organisms in the hypodermal chords (arrows). Scale bars: A and B = 20 μm; C = 5 μm; D = 10 μm.

Reactivity for WSP was positive in the hypodermis of *O. armillata* adult female worms (aorta sections from different animals; *n* = 4) and in the positive control, *O. ochengi* (Fig. 1). Furthermore, *Wolbachia* could also be detected in *O. armillata* Mf contained within the female reproductive tract (Fig. 1C). The *O. armillata* worms within the single nodule examined appeared to be dead and did not stain distinctly for WSP.

### 3.5 PCR of *O. armillata* DNA extract

**Fig. 2:**
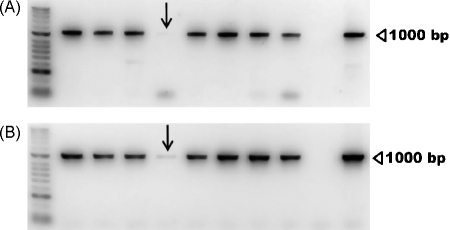
1% agarose gels loaded with PCR reactions for *Wolbachia ftsZ* (A) and the *Wolbachia* 16S rRNA gene (B). Lanes 2–4 (from left) and 6–9 show PCR products for *O. armillata* adult female worm DNA extracts (≥5 individuals); lane 5 represents a DNA extract from a single male worm (arrow). Lane 10, negative control (water); lane 11, positive control (*O. ochengi* DNA extract); lane 1, HyperLadder™ II molecular weight marker (Bioline). The images have been black-white inverted for clarity.

The PCR assays on the DNA extracted from adult worms showed *O. armillata* to be positive for both the *Wolbachia ftsZ* gene and the *Wolbachia* 16S rRNA gene (Fig. 2). The expected DNA product of approximately 1000 bp was present in the reactions for both primer pairs (16SWolbF/16SWolbR3 and ftsZfl/ftsZrl) for all adult female worm extracts (*n* = 50; female worms were screened in pools of ≥5 individuals from different host animals). The single male worm analysed was positive for both genes, but with a very weak signal, especially for the *ftsZ* gene.

### 3.6 Pathology

**Fig. 3:**
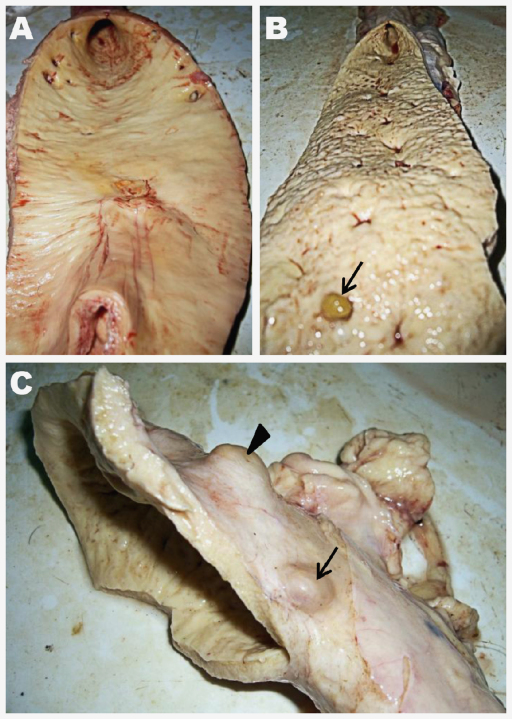
Gross pathology of *O. armillata* infection in the aorta. The intimal surface of the aortic trunk from a 4-year-old cow (A) can be compared with that of an 8-year-old female (B). An *O. armillata* nodule is visible in the foreground (B, arrow) extending into the aortic lumen of the older cow. In (C), the aortic arch and associated serosa of an 8-year-old cow (same subject as in B) exhibits both calcified (arrowhead) and caseous (arrow) nodules that extend from the tunica media into the tunica adventitia.

In mild infections, the aortic intima appeared smooth with the occasional small (up to 1 cm diameter), uncalcified nodule (Fig. 3A). Milder infections, usually seen in younger animals, were confined to the region of the aortic arch. Yellow-brown tortuous tunnels that were usually slightly raised were present under the tunica intima. It was found that a small proportion of these tunnels contained no worm. Heavier burdens of parasite infection resulted in thicker and less elastic aortic walls (Fig. 3B) and an uneven intimal surface with more numerous nodules, many of which were calcified. Heavier infections also showed spread to more caudal parts of the aorta.

In putatively chronic cases, nodules were found additionally on the tunica adventitia (up to 2.5 cm diameter). Some of these nodules resembled lymph nodes (Fig. 3C). Older, larger nodules were capsular and contained calcified material and/or a creamy yellow, caseous exudate. On a few occasions, marked atrophy was apparent where a large, well-defined nodule spanned the majority of the aorta wall originating from the tunica media. No parasites were found free, or partially free, in the lumen of the vessel.

### 3.7 Histopathology

**Fig. 4:**
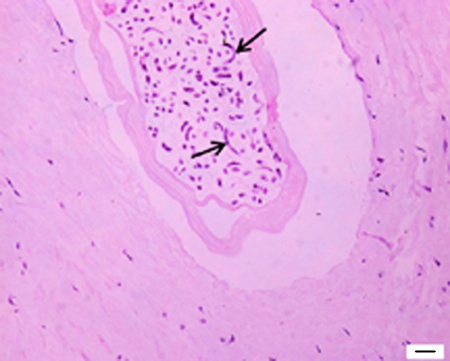
An adult female *O. armillata* residing within a tunnel inside the tunica media of the aorta. No inflammatory cells are present. The uterus of the worm contains mature Mf (arrows). Scale bar: 20 μm.

Microscopically lesions due to worm presence were principally found in the tunica media, but some encroached the intima and, in chronic infections, the adventitia. Cell responses ranged from no or few inflammatory cells in the early stage (Fig. 4), to high numbers of granulocytes, macrophages, fibroblasts and multinucleate giant cells in older lesions. A degenerating worm with a surrounding inflammatory infiltrate could be found in the same histological section as a viable worm with no inflammatory response or an empty tunnel. This suggests that individual hosts are repeatedly re-infected.

Worms appeared to reside within a cavity, with a space between the worm section and the host-derived lining. Factor VIII-related antigen staining (data not shown) showed this lining not to be composed of endothelia, but rather to be continuous with the tunica media.

**Fig. 5:**
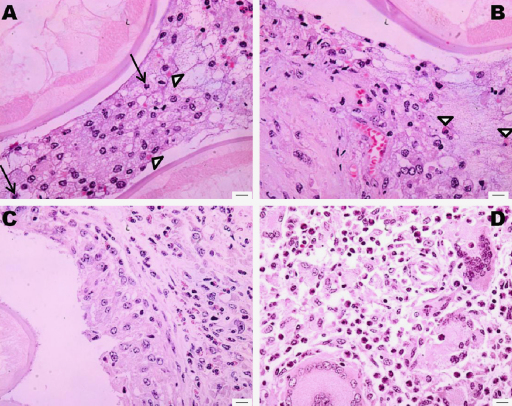
Inflammatory response to *O. armillata*. (A) Macrophages separate two cross-sections of adult worms. The foamy cytoplasm of the macrophages suggests that they are actively phagocytosing material. Neutrophils (arrows) and extravasated erythrocytes (arrowheads) are present in low numbers. (B) Foamy macrophages with extensive fat-filled cytoplasm surround the worm cuticle. Many of the foamy macrophages have undergone necrosis. Small numbers of eosinophils can be seen in the periphery (arrowheads). Note the area of vascular injury (lower centre). (C) Granulomatous response to an adult worm. Macrophages up to several cells thick line the cavity. This is surrounded by a cuff of fibroblasts and more peripherally, granulocytes. (D) Multinucleate giant cells, macrophages and neutrophils within the tunica media (no parasite visible). Scale bars: 10 μm.

Degenerate, dead and calcified worms caused a more marked inflammatory response consisting predominantly of macrophages (Fig. 5A). Typically, macrophages of one or several layers occupied the region closest to the worm cuticle, with small numbers of eosinophils (Fig. 5B) and neutrophils (Fig. 5A) more peripherally. Chronic inflammation (composed of lymphocytes, plasma cells and multinucleate giant cells) characterised the most peripheral aspect of the local immune response (Fig. 5C). Fibroblasts and collagen were interspersed amongst the inflammatory cells. A thin capsule of circularly arranged fibrous tissue circumscribing the cavity was apparent in older lesions (granuloma formation). Microvasculature was apparent in some capsules, but not all, and evidence of vascular injury was present in several specimens (Fig. 5B).

Large multinucleate giant cells were often found in regions of the media not occupied by a parasite (Fig. 5D) and perivascularly. Inflammatory cells were never found adherent to the worm cuticle, and no degranulation onto the cuticle was observed. Prussian blue staining did not reveal the presence of haemosiderin within the gut of worm sections; and von Kossa staining did not detect tissue mineralization in the samples examined (data not shown).

## 4 Discussion

The majority of filarial nematodes have been found to contain the endosymbiont *Wolbachia*. It has been suggested that *Wolbachia* may be important in evading the host immune response in those species of *Onchocerca* associated with the bacteria (Brattig et al., 2001; Nfon et al., 2006).

In the present study, *O. armillata* was found to contain *Wolbachia* in the hypodermis of adult female worms and in intrauterine Mf using immunohistochemical techniques. This was verified by PCR amplification of *Wolbachia*-associated genes. It has recently been reported that *Onchocerca dewittei japonica* (a parasite of wild boar) only harbours *Wolbachia* in the female reproductive tract, with an absence of bacteria in the female hypodermis and in male individuals (Bain et al., 2008). In addition, it appears that some *Onchocerca* spp. exhibit polymorphism for the presence of *Wolbachia* (Bain et al., 2008). For these reasons, and because the adult worms were often damaged after extraction from the aorta, we performed PCR analyses on pools of several adult females to maximise the probability of detecting infection. This approach did not allow us to determine the probability that *Wolbachia* infection is fixed in this population of *O. armillata*, and due to the difficulty of obtaining specimens we did not attempt to identify the location of *Wolbachia* in the tissues of male worms. However, the key question arising from this study is the role of *Wolbachia* (if any) in the evasion of the bovine immune response by *O. armillata*.

The cellular response to *O. armillata* appeared to be less intensive with fewer granulocytes, particularly neutrophils, when compared to *O. volvulus* and *O. ochengi*. As previously observed by Ogundipe et al. (1984), many viable worms had little or no surrounding inflammatory response. Only degenerating, dead or calcified worms in the nodules or aorta wall were associated with a chronic granulomatous response. The prevalence of eosinophils increased with the age of the lesion as noted in several other studies (Chodnik, 1957; Schillhorn van Veen and Robl, 1975; Atta el Mannan et al., 1984; Ogundipe et al., 1984; Mtei and Sanga, 1990), but not to the same degree as reported for other *Onchocerca* spp. A response dominated by multinucleate giant cells was only evident in cattle older than 5 years. This suggests a lifespan for *O. armillata* of at least this duration.

The role of motility in the evasion of the immune response by filariae and other tissue-dwelling nematodes has been recognised for decades, although the focus has been on the larval stages, which are easier to study *in vitro*. For instance, Sim et al. (1982) demonstrated the clear association between loss of motility and adherence of leukocytes after incubation of *B. malayi* L3 with human immune serum; and it is well established that the microfilaricidal drugs ivermectin and diethylcarbamazine exert their effects (at least in part) by impeding the motility of microfilariae, thus facilitating the attachment of host effector cells and destruction of the parasites in the lymph nodes (Racz et al., 1982; Darge et al., 1991).

For adult filariae, at least three different evolutionary strategies appear to have been employed to avoid the inflammatory response of the mammalian host. Firstly, there are motile species that may either migrate through the host's tissues leaving a trail of inflammatory cells in their wake that fail to circumscribe the parasite [such as the *Wolbachia*-negative *Loa loa* (Gutierrez, 2000)]; or which may otherwise ‘dance’ within the confined environment of a cystic nodule [*e.g. O. jakutensis* (Plenge-Bönig et al., 1995)] or worm nest [*e.g. W. bancrofti* (Norões et al., 1997)], apparently preventing the accumulation of leukocytes on the worms’ surface. In these species, there is no evidence that *Wolbachia* is involved in immune evasion, and its role may ‘simply’ be metabolic provisioning, although clearly there are filarial taxa that thrive without it. Secondly, the *Onchocerca* spp. with degenerated musculature in the female and a sessile lifestyle in fibrous nodules (*O. ochengi*, *O. volvulus* and *Onchocerca gibsoni*) may depend on a *Wolbachia*-mediated “immunological blockade”, comprising a local neutrophilia that interferes with eosinophil infiltration and degranulation (Nfon et al., 2006). The only known *Wolbachia*-negative *Onchocerca* spp., *O. flexuosa*, may utilise a third strategy as the females are sessile in fibrous nodules, yet do not invoke a neutrophilic response (Brattig et al., 2001). Evidence from partial genome sequencing has demonstrated that this species once harboured *Wolbachia* (McNulty et al., 2010). One mechanism by which it may have compensated for loss of the endosymbiont is to have accelerated its development to sexual maturity, as it appears to have a much shorter lifespan than the *Wolbachia*-positive nodular species (Plenge-Bönig et al., 1995).

Females of *Onchocerca* spp. that do not form nodules and which have retained a well-developed somatic musculature [*e.g. O. gutturosa* (Franz et al., 1987)] are probably not as active as *L. loa*, but nevertheless, may retain the ability to dislodge host effector cells by sloughing and thus express a variation of the first strategy. Chodnik (1957) suggested that *O. armillata* is a motile species due to the many vacant tunnels within histological sections. This is in accordance with the histological observations and the experience of manual extraction of adult worms from the aorta wall in our study. Furthermore, the musculature of adult *O. armillata* female worms is prominent (Franz et al., 1987), and the immunological reaction described in the current study (*i.e.*, dominated by macrophages and giant cells, with small numbers of granulocytes more distant) is similar to that reported for *O. gutturosa*, although it may be less intense (Wildenburg et al., 1997). The vascular injury noted here and also by Chodnik (1957) provides further support for a nomadic lifestyle for *O. armillata*. However, definite evidence to support this hypothesis has not been obtained, as it would be extremely difficult to visualise adult worms *in vivo* in the deep anatomical location of the aorta.

The high prevalence (90.7%) of *O. armillata* reported in this study and the severity of the pathological lesions may have implications for animal production in the tropics. Similarly high infection rates of 99% (Patnaik, 1962), 86% (Alibasoglu et al., 1969), 92% (al-Zubaidy, 1973) and 95.4% (Mtei and Sanga, 1990) have been reported in India (Orissa State), Turkey, Iraq, and Tanzania, respectively. Although no obvious signs of illness are reported in animals infected with *O. armillata*, the severity of the pathological lesions described must reduce the efficiency of blood flow through the aortic arch. The inflammatory response in the tunica media with destruction of the muscular structure could result in weakening of the aorta wall. On its own, this parasite may have little veterinary importance, but in combination with the multiple parasitic infections that many cattle harbour in the region, the cumulative effects may be significant. Previous authors have speculated that *O. armillata* adult worms to be the cause of aneurysmal cardio-vascular disturbances (Nelson, 1970) and aortopathy (Zak, 1975).

The skin-dwelling Mf of *O. armillata* may be uniformly distributed throughout the skin (Wahl et al., 1994) or, as reported in other studies (Elbihari and Hussein, 1976; Atta el Mannan et al., 1984), agglomerate in the region of the hump. This may reflect variation in biting behaviours of the vector in different areas and/or the possibility of different vectors distributed across the parasite's wide geographic range. *O. ochengi* Mf were located in greatest abundance ventrally, which accords with the preferred feeding site of its vector (Wahl and Renz, 1991). In the current study, the hump appeared to be the site most favoured by *O. gutturosa*, as reported previously (Elbihari and Hussein, 1978).

Although not significant in this study, a similar reduction in the density of *O. armillata* Mf within the epidermis of older cattle has been previously observed (Atta el Mannan et al., 1984). This trend may be due to an increase in old, less fecund or dead, calcified worms in older cattle (Trees et al., 1992); and/or a degree of immunity in hyper-endemic areas may be acquired, which has been found to occur against *O. ochengi* Mf (Trees et al., 1992).

Younger cattle, having had less exposure to the biting vectors, would be expected to have a lower prevalence of infection than the high level (100%) found in this study. Further investigation is required to establish the identity of the biological vector for *O. armillata*, and how it is so highly successful in transmitting the infection.

## 5 Conclusion

This study provides the first evidence that *O. armillata* contains the endosymbiotic bacterium, *Wolbachia*. If, as previously supposed, the neutrophil chemotactic activity in filarial nematodes is largely dependent on the presence of *Wolbachia* (Nfon et al., 2006), the cellular response to adult *O. armillata* worms should primarily consist of these cells. However, in contrast to *O. ochengi*, a heavy concentration of neutrophils around adult worms was not observed. Indeed, granulocytes were a relatively small component of the cellular infiltration, which was dominated by macrophages and fibroblasts in close proximity to the worm, with small clusters of eosinophils peripherally. Thus, *O. armillata* appears to be protected from eosinophil degranulation, but the mechanism involved for this putatively motile species may differ from that observed in nodule-forming *Onchocerca* spp.

## Conflict of interest statement

None of the authors declares any conflict of interest.

## Figures and Tables

**Table 1:** Prevalence and density of *Onchocerca armillata* infection by age group in cattle slaughtered at Ngaoundéré abattoir.

	Age category (years)				
	≤3	4–5	6–7	≥8	*P*
No. examined	8	24	15	7	–
No. with adult worms	8	21	13	7	0.78^a^
No. with patent infection^b^	2	7	2	1	0.68^a^
Geometric mean Mf 100 mg^−1^ skin^c^	0.15	0.29	0.13	0.06	0.54^d^
No. with aortic nodules	5	13	10	4	0.78^a^

a Fisher's exact test.

b *i.e*., no. with Mf in skin snips.

c Group geometric means were calculated from the arithmetic means of the single hump and ventral skin sample counts per animal. Zero counts were included by *x* + 1 transformation.

d Kruskall–Wallis test.

**Table 2:** Prevalence of *Onchocerca* spp. in cattle slaughtered at Ngaoundéré abattoir, as assessed by presence of microfilariae in skin snips (*n* = 50).

Species	Prevalence (%)	Mf density 100 mg skin^−1^ at sampling site (geometric mean)^a^	
		Hump	Ventral midline
*O. gutturosa*	100	35.5	23.9
*O. ochengi*	66	3.9	19.6
*O. armillata*	25	1.8	1.3
*O. dukei*	4	7.0	2.0

a As only patent infection was recorded for *Onchocerca* spp. other than *O. armillata*, data exclude Mf counts of zero to aid interpretation.
